# Analysis of clinical features and genetic variants in Chinese children with pyridoxine-dependent epilepsy: a case series study

**DOI:** 10.3389/fneur.2025.1609600

**Published:** 2025-09-04

**Authors:** Xixi Yu, Xin Zhang, Shiyan Qiu, Li Yang, Xin Li, Dongyu Shi

**Affiliations:** ^1^School of Clinical Medicine, Shandong Second Medical University, Weifang, China; ^2^Department of Pediatric Neurology, Linyi People’s Hospital, Linyi, China

**Keywords:** pyridoxine, epilepsy, child, mutation, *ALDH7A1* gene

## Abstract

**Objective:**

To summarize the clinical features and the spectrum of *ALDH7A1* gene variants in Chinese children with pyridoxine-dependent epilepsy (PDE).

**Methods:**

Clinical data were collected from six pediatric patients with PDE treated at Linyi People’s Hospital (2017–2023). Whole-exome sequencing, in conjunction with Sanger sequencing, was used to screen for *ALDH7A1* gene variant sites. Bioinformatics methods were applied in combination with clinical phenotype assessment to evaluate the pathogenicity of the variants.

**Results:**

In six cases of PDE, the male-to-female ratio was 2:4. Five cases began in the neonatal period, and one in early infancy. Three cases experienced hypoxiaetal distress or respiratory distress. Two cases had a family history of recurrent miscarriages or premature deaths of siblings due to seizures. Seizure types varied among the cases. Three individuals experienced cluster seizures, two were heat-sensitive, and four suffered from status epilepticus. Video EEGs of four children revealed hypsarrhythmia, focal, or multifocal discharges initially, which normalized within 3 days to 8 months following vitamin B6 treatment. All patients initiated regular vitamin B6 therapy between 32 days and 17 months post-onset, with an initial dose ranging from 7.7 to 12.5 mg/(kg·d). During follow-up until the age of 1 to 8 years, the maintenance dose was adjusted to 3.3–7.8 mg/(kg·d). Two patients who began standardized treatment before the age of 3 months experienced normal development. The remaining patients showed mild to moderate developmental delays. Eight variants were identified, including a hotspot in Chinese children: c.1008+1G>A, and two recurrent sites: c.1061A>G and c.1547A>G. Additionally, three variants were novel.

**Conclusion:**

Pyridoxine-dependent epilepsy must be distinguished from hypoxic–ischemic encephalopathy and other thermally sensitive epilepsies. The severity of the condition and neurodevelopmental outcomes in affected children vary significantly. Early administration of low-dose pyridoxine can effectively control seizures and reverse EEG abnormalities, potentially improving long-term neurodevelopmental outcomes. c.1061A>G and c.1547A>G may be hotspot variants in Chinese children with PDE.

## 1 Introduction

Pyridoxine-dependent epilepsy (PDE) is a rare autosomal recessive disorder first reported by Hunt et al. (1). The disease is characterized by an early age of onset, with refractory seizures beginning in neonates and early infancy, poor response with conventional antiseizure medication and effective treatment with pyridoxine (2). In 2006, *ALDH7A1* was identified as the pathogenic gene for PDE (3). In 2022, the International League Against Epilepsy (ILAE) classified PDE as a cause-specific epilepsy syndrome and named it pyridoxine dependent (*ALDH7A1*) – developmental and epileptic encephalopathies, PD-DDE (4). PDE is an epileptic syndrome associated with defects in the lysine metabolic pathway, with a prevalence rate of 1:64,352 in live births (5). To date, a total of 225 *ALDH7A1* gene variant sites have been reported both domestically and internationally, with IVS11+1G>A being a hotspot variant site among Chinese children (6).

Approximately one-third of patients with PDE exhibit atypical clinical presentations. These patients experience the onset of their disease from infancy to adolescence. Initially, the condition may be well managed with conventional antiseizure medication or prove resistant to early trials of vitamin B6. Some patients may experience prolonged seizure-free periods following the cessation of vitamin B6 treatment (7). Toehold et al. have also identified pathogenic *ALDH7A1* variants in some patients with developmental malformations of the brain (8). Atypical clinical features complicate the diagnosis of PDE, and delayed administration of pyridoxine therapy is associated with more severe neurological damage (9). In recent years, researchers have proposed methods to detect *α*-AASA, P6C, and PA concentrations using dried blood spots and dried urine spots (10, 11). These methods have shown potential for screening for PDE, but they have not yet been widely adopted or applied in clinical practice. Currently, genetic testing remains the primary method for diagnosing the disease.

Limited by the number of cases, the clinical features and gene variant characteristics of PDE have not been comprehensively summarized. Additionally, the case data of some studies were sourced from open databases, and it remains challenging to predict the severity of the associated diseases without detailed clinical information. In this study, we employed second-generation sequencing technology to detect variants of the *ALDH7A1* gene, with the aim of summarizing the clinical manifestations and prognostic characteristics of children with PDE. Additionally, we sought to identify potential hotspot variant sites and those previously unreported, with the goal of providing clinicians with a reference for the early identification and diagnosis of this disease.

## 2 Methods

### 2.1 Clinical data collection

Six pediatric patients diagnosed with PDE at Linyi People’s Hospital between January 2017 and December 2023 were selected as the study subjects. A clinical registry was established, encompassing details such as name, gender, age of onset, predisposing factors, seizure type, seizure frequency and duration, medication history, perinatal condition, neurodevelopment, family history of epilepsy, video electroencephalogram (VEEG), cranial magnetic resonance imaging (MRI), and blood and urine metabolic screening. The children’s progress was monitored via routine outpatient visits and phone calls to track adjustments to their medication, epilepsy control, VEEG changes, and neurodevelopmental outcomes later in life. The study received review and approval from the Ethics Committee of Linyi People’s Hospital (13003), and all guardians of the children provided informed consent by signing a form.

### 2.2 Genetic testing and pathogenicity analyses

Anticoagulation tubes with Ethylene Diamine Tetraacetic Acid (EDTA) were used to collect 2 mL of peripheral blood samples from each affected child and their primary family members. Genomic DNA was extracted using the QIAamp Whole Blood DNA Kit (Qiagen, Germany), and whole exome sequencing was conducted. The sequencing data were compared to the human reference genome (hg38 version) using BWA software. Single nucleotide variants and insertion/deletion variants were analyzed using GATK software. The frequencies of variant sites in the Exome Aggregation Consortium (ExAC), Genome Aggregation Database (gnomAD), and 1,000 Genomes Project (1000G) were queried. Protein structure prediction software, including SIFT, Mutation Taster, and GERP++, was used to predict the deleterious properties of variants. Variants were analyzed for multiple homologous direct biological conservatisms. Protein structure prediction of variant expression was performed using Swiss-Model software. Protein structure visualization analysis was conducted using PyMOL software. The pathogenicity of the variants was assessed in accordance with the American College of Medical Genetics (ACMG) guidelines (12). Sanger sequencing was carried out to validate the suspected pathogenic variant sites.

## 3 Results

### 3.1 General information

Among the six cases of PDE, there were two males and four females. The onset age ranged from 1 day to 5 months, with a median of 6 days. Five cases experienced onset during the neonatal period. Three cases experienced fetal distress or respiratory distress. Throughout the course of the disease, all six patients presented with multiple seizure types, including tonic seizures in two cases, focal tonic seizures that evolved into focal or bilateral clonic seizures in three cases, epileptic spasms in three cases, and clonic seizures in two cases. Three cases (50.0%) exhibited cluster seizure features, two cases (33.3%) had heat sensitivity, and four cases (66.7%) had experienced status epilepticus lasting between one to 3 h.

### 3.2 Auxiliary examinations

At the onset of the disease, the interictal VEEG revealed hypsarrhythmia in one case, multifocal discharges in two cases, and normal or borderline findings in three cases. Among these, case 3 initially presented with a normal VEEG in the first month after onset. However, upon review, focal or multifocal discharges emerged in the second to third month post-onset. Of the six patients with PDE, two had interictal VEEG results that were normal or borderline before and after vitamin B6 treatment, while four VEEG recordings normalized within 3 days to 8 months following consistent vitamin B6 treatment. All six cases underwent cranial MRI examinations, and three were found to be normal. Cases 3 and 4 exhibited subarachnoid enlargement, whereas case 6 presented with enlarged lateral ventricles and large occipital pools, as detailed in Table 1.

**Table 1:** The clinical manifestations with PDE.

No.	Sex	Birth history	Onset age of epilepsy (m.d)	Initial seizure types	Interictal VEEG	MRI
1	F	Fetal distress	0.5	Epileptic spasms	Multifocal discharges (12d) → normal (40d)	Normal
2	F	Respiratory distress.	5	1. Focal tonic. 2. Focal tonic evolving into bilateral clonic	borderline or normal	Normal
3	M	N	0.3	Focal clonic	Normal (46d) → focal or multifocal discharges (68d/3 m) → normal (7 m)	Normal
4	F	N	0.17	1. Focal tonic 2. Focal tonic evolve into focal clonic 3. Epileptic spasms	Hypsarrhythmia (3 m) → normal (13 m)	Subarachnoid enlargement
5	F	N	0.7	Focal tonic evolve into bilateral clonic	Normal	Subarachnoid enlargement
6	M	Respiratory distress.	0.3	1. Focal clonic 2. Epileptic spasms	Multifocal discharges (31d) → normal (10 m)	Enlarged lateral ventricles and large occipital pools

F, female; M, male; N, normal; d, days; m, months.

### 3.3 Treatment

Six children were treated with one to four conventional antiseizure medication following the onset of their disease, all of which proved ineffective or only temporarily effective. They began regular application of vitamin B6 from day 32 to 17 months after the onset, as detailed in Table 2. Four cases (4/6, 66.7%) were hospitalized to receive intravenous vitamin B6 during periods of frequent convulsions, and all were seizure-free during the treatment period. Case 1 was treated with intravenous vitamin B6 at a dose of 13.5 mg/(kg·d) in combination with topiramate for 6 days, beginning on day 5 post-onset. Following discharge from the hospital, the patient was administered topiramate alone. Due to recurrent convulsive episodes, regular oral vitamin B6 was started on day 32 post-onset. On day 48 post-onset, the genetic report was received, leading to the subsequent reduction and discontinuation of topiramate. In Case 2, the patient was hospitalized multiple times at the onset of the disease and treated with intravenous vitamin B6 at a dose of 10.0 mg/(kg·d). During this period, the dosage and type of regular antiseizure medication were also adjusted, and vitamin B6 was discontinued post-hospital discharge. She began taking regular vitamin B6 supplements in the 17th month following the onset of the disease, and her genetic report was received in the 18th month after the onset, followed by a gradual withdrawal of other drugs.

**Table 2:** Treatment options and follow-up for PDE.

No.	Age of regular oral vitamin B6	Initial dose of vitamin B6	Previous AEDS	The last follow-up		
				Current age	Current dose of vitamin B6	Development
1	37d	8.9 mg/kg	PB/TPM/VitB6	8y	3.3 mg/kg/d	Normal
2	22 m	12.5 mg/kg	LEV/VPA/TPM/CZP/VitB6	4y	7.8 mg/kg/d	moderate Cognitive, gross motor and language backwardness
3	3 m	7.7 mg/kg	LEV/VitB6	5y	5.2 mg/kg/d	Normal
4	6 m	11.5 mg/kg	LEV/VPA/VitB6	6y	4.9 mg/kg/d	Mild cognitive backwardness
5	6 m	11.8 mg/kg	TPM/VitB6	5y	6.3 mg/kg/d	Mild cognitive and gross motor backwardness
6	2 m	10.9 mg/kg	PB/LEV/VitB6	1y	6.9 mg/kg/d	Mild language backwardness

m, months; d, days; y, years; PB, phenobarbital; TPM, topiramate; LEV, levetiracetam; VPA, valproic acid; CZP, Carbamazepine.

### 3.4 Prognosis

During the last follow-up, the patients’ ages ranged from 1 to 8 years old. All patients experienced significant relief from seizures. The duration of relief varied from 1 to 23 months, with a median of 4.5 months. Only Case 2 occasionally experienced seizures during a fever. The maintenance dose of vitamin B6 ranges from 3.3 to 7.8 mg/(kg·d). Two cases exhibited normal growth, while four cases showed mild to moderate developmental delay, as shown in Table 2. Among them, Case 2 exhibited normal neurodevelopment before the disease onset but experienced developmental arrest subsequently. By the age of two, she was still unable to walk, speak, or recognize her family. However, by the age of four, she had made significant progress, managing to speak 3 to 5 words, walk steadily, and recognize her parents.

### 3.5 Genetic analyses

**Figure 1:**
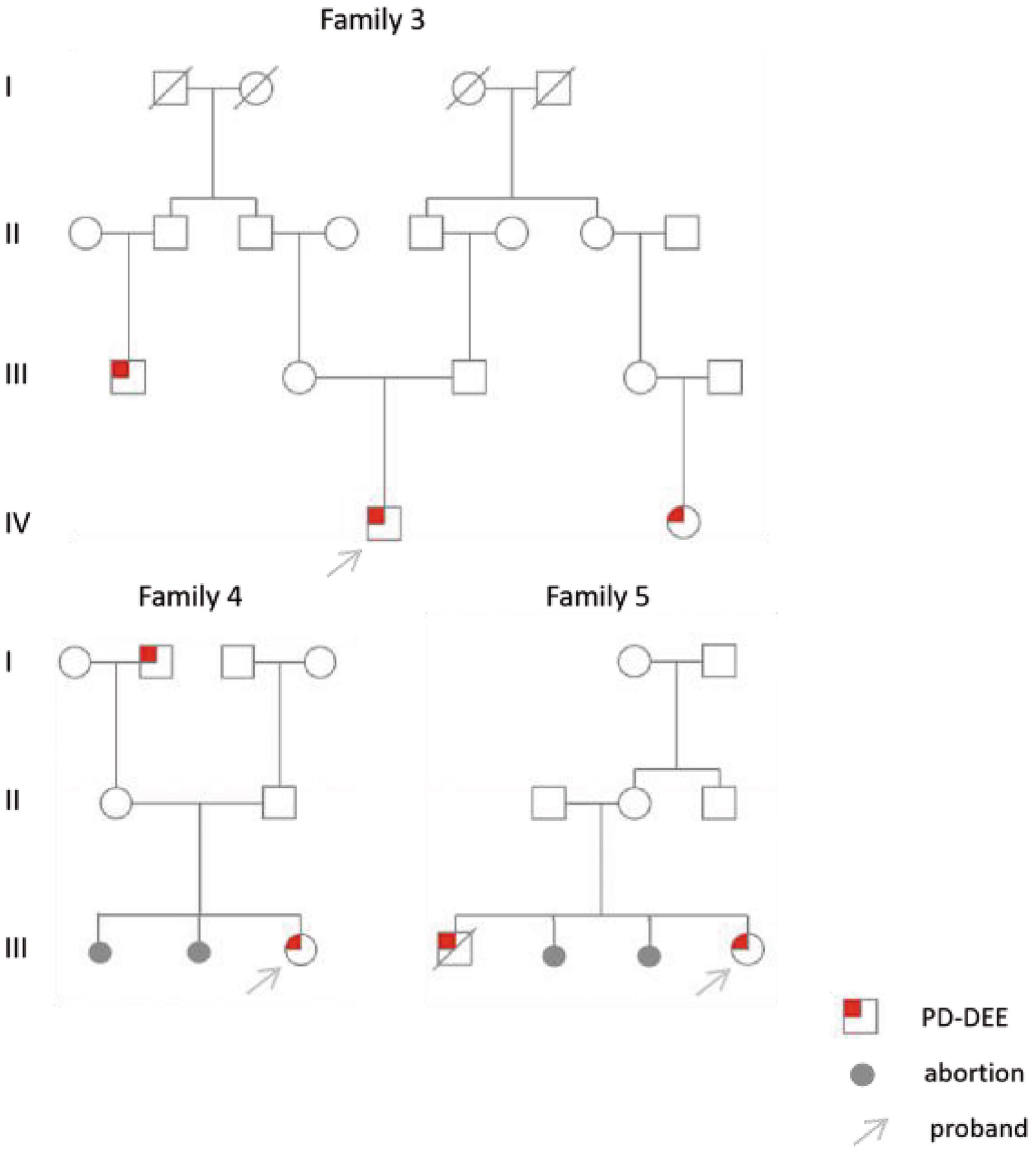
Family chart of three children.

**Figure 2:**
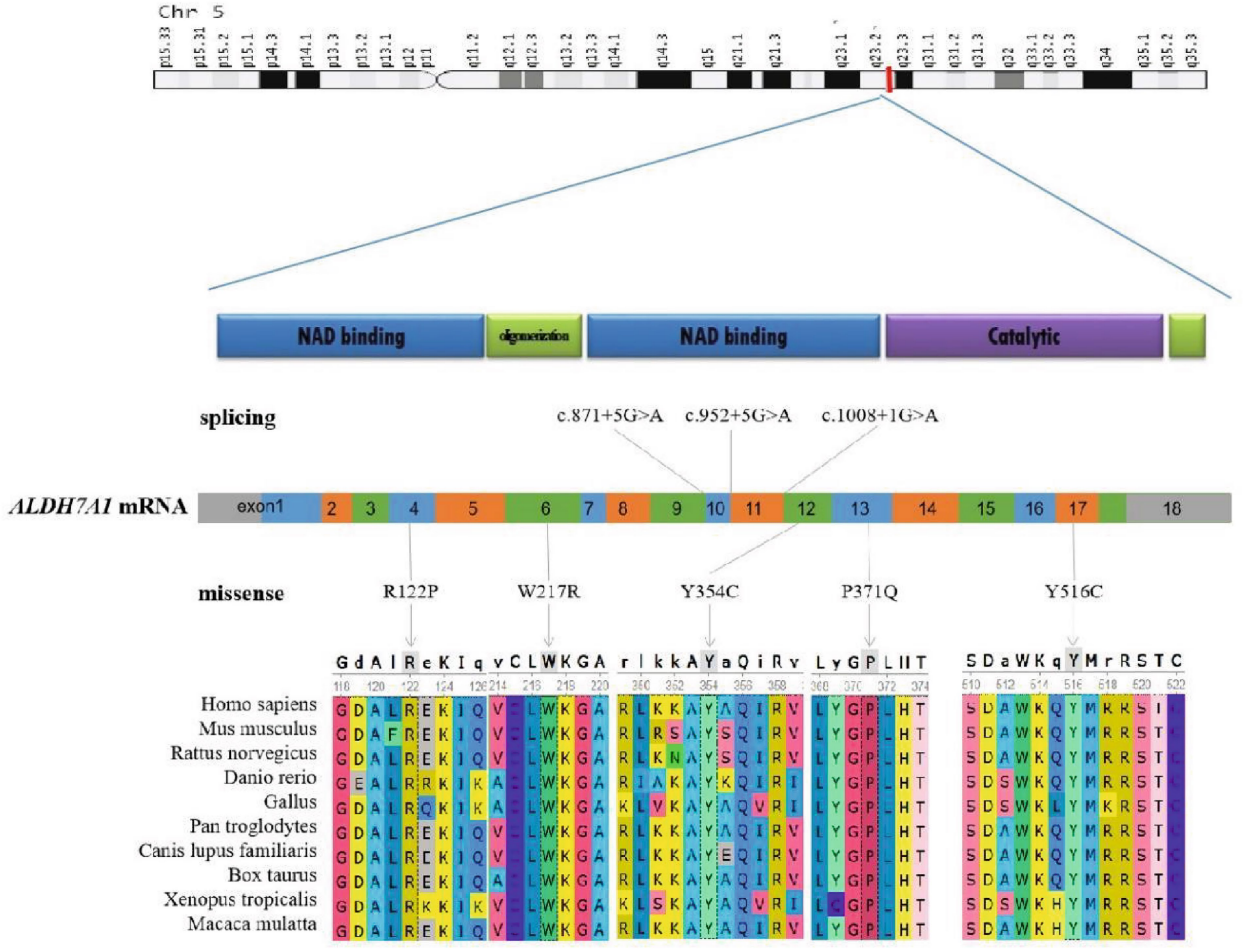
Schematic representation of the *ALDH7A1* variants. The two-dimensional structure of the *ALDH7A1* protein. The positions of splicing variants and missense variants on the chromosome and mRNA counterparts. Conservation of five missense variant sites in humans, *Mus musculus*, *Rattus norvegicus*, *Danio rerio*, Gallus, *Pan troglodytes*, *Canis lupus familiaris*, *Bos taurus*, *Xenopus tropicalis*, and *Macaca mulatta*.

**Figure 3:**
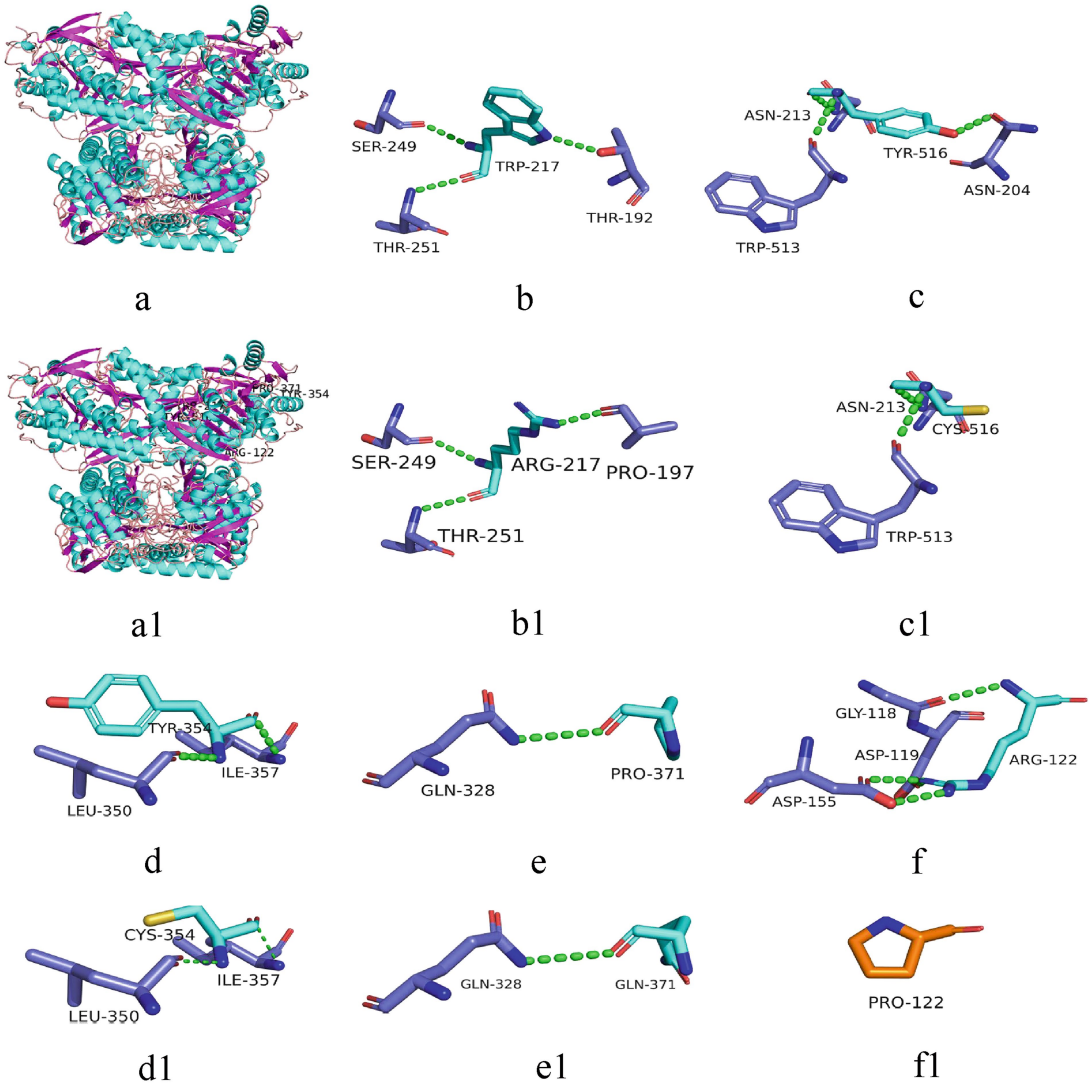
3D structure of the protein associated with *ALDH7A1* variants. The 3D structures of proteins corresponding to five missense variants are depicted. a: 3D structure of the wild-type protein; a1: corresponding positions of the five variant sites on the protein; b-f: 217Trp, 516Tyr, 354Tyr, 371Pro, 122Arg and their surrounding hydrogen bonds and peptide chains in the wild-types; b1-f1: 217Arg, 516Cys, 354Cys, 371Gln, 122Pro and their surrounding hydrogen bonds and peptide chains in the variants. Green dashed lines represent hydrogen bonds.

All six cases carried compound heterozygous variations in the *ALDH7A1* gene, and three of these cases (50.00%) had a family history of epilepsy, as illustrated in Figure 1. A total of eight gene variation sites were identified. The pathogenicity of these variations was assessed following the ACMG guidelines, with the outcomes presented in Table 3. Notably, the variation site c.1061A>G was found to be repeated three times, while the variation site c.1547A>G occurred twice. A hotspot variant site in Chinese children with PDE was identified in two cases: IVS11+1G>A. The remaining three variant sites (3/8, 37.5%) were internationally unreported: c.649T>A, c.1112C>A, c.365G>C. Among the eight variants, five (5/8, 62.50%) were missense variants, which are highly conserved across various species, including humans, house mice, brown rats, zebrafish, chickens, and chimpanzees. Three (3/8, 37.50%) of the variants were splicing variants, as depicted in Figure 2. Three cases (3/6, 50.00%) had a family history of epilepsy, as illustrated in Figure 1. Figure 3 displays the 3D structures of the proteins corresponding to the five missense variants. The changes at the 217th, 516th, and 122nd amino acid residues resulted in significant alterations to the surrounding hydrogen bonds. Alterations at the 354th and 371st amino acid residues led to changes in the protein’s peptide chain structure. These variations could impact the stability and function of the protein’s three-dimensional structure. Six pediatric patients displayed characteristics of autosomal recessive inheritance. Based on their clinical symptoms, they were diagnosed with PDE.

**Table 3:** Pathogenicity analysis of PDE associated with *ALDH7A1* gene variations.

No.	Source	Nucleotides	Amino acids	SIFT	Mutation Taster	GERP++	gnomAD	ExAC_E	1000G	ACMG scoring	ACMG pathogenicity	Reported or not
1	F	c.649T>A	p. W217R	D	D	C	0	0	0	PM2 + PM3 + PP3	VUS	N
	M	c.1547A>G	p. Y516C	D	D	C	6.46E-05	6.59E-05	0.000199681	PS1 + PM2 + PM3 + PP3	LP	PMID: 35,495,162
2	F	c.1061A>G	p. Y354C	D	D	C	0	0	0	PS1 + PM2 + PM3 + PP3	LP	PMID: 22371912
	M	c.1008+1G>A	splicing							PVS1 + PS1 + PM2 + PM3	P	PMID: 24664145
3	F	c.1061A>G	p. Y354C	D	D	C	0	0	0	PS1 + PM2 + PM3 + PP3	LP	PMID: 22,371,912
	M	c.1112C>A	P371Q	D	D	C	0	0	0	PM2 + PM3 + PM5 + PP3	LP	N, PMID: 34,426,522
4	F	c.365G>C	p. R122P	D	D	C	6.46E-05	0	0	PM2 + PM3 + PP3	VUS	N
	M	c.952+5G>A	splicing							PVS1 + PS1 + PM2	P	PMID: 26,555,630
5	F	c.1547A>G	p. Y516C	D	D	C	6.46E-05	6.59E-05	0.000199681	PS1 + PM2 + PM3 + PP3	LP	PMID: 35495162
	M	c.1008+1G > A	Splicing							PVS1 + PS1 + PM2 + PM3	P	PMID: 24664145
6	F	c.1061A>G	p. Y354C	D	D	C	0	0	0	PS1 + PM2 + PM3 + PP3	LP	PMID: 22371912
	M	c.871+5G>A	splicing							PS1 + PM2 + PM3	LP	PMID: 26555630

M, mother; F, father; SIFT, (D, damaging; T, tolerabled); Mutation Taster mutation (D, disease_causing); GERP++, (C, conserved); P, pathogenic; LP, likely pathogenic; VUS, variants of uncertain significance.

## 4 Discussion

The PDE is a rare autosomal recessive genetic disorder primarily caused by variations in the *ALDH7A1* gene. This gene is located on the long arm of chromosome 5 at position 31 (5q31) and spans a total length of 53,550 base pairs (bp). The enzyme it encodes, α-aminoadipic semialdehyde dehydrogenase, is involved in the final step of lysine catabolism: the oxidation of α-aminoadipic semialdehyde (α-AASA) to α-aminoadipic acid (α-AA) (13). Dysfunction of the *ALDH7A1* gene results in reduced activity of the enzyme, causing the accumulation of α-AASA and its cyclized form, Δ1-piperidine-6-carboxylate (P6C), within the body. Both α-AASA and P6C exhibit neurotoxic properties (14). Additionally, the accumulated P6C binds to pyridoxal 5′-phosphate (PLP), thereby inactivating it. PLP, the primary active form of vitamin B6, plays a crucial role in neurotransmitter metabolism. Its inactivation disrupts the synthesis of various neurotransmitters, particularly the inhibitory neurotransmitter gamma-aminobutyric acid (GABA) (15). These mechanisms collectively lead to recurrent epileptic seizures in children with PDE. From a pathophysiological perspective, supplementation with vitamin B6 can increase PLP concentrations in the body, thereby controlling epileptic seizures.

Presently, 225 variants of the *ALDH7A1* gene have been reported globally. These encompass 109 missense variants (48.4%), 19 nonsense variants (8.4%), 35 splicing variants (15.6%), and 62 small insertions/deletions (27.6%). Among them, missense variants constitute the highest proportion. Population-specific analysis has revealed that c.1279G>C is a hotspot variant among European PDE patients, with a carrier frequency of approximately 30% (16). Meanwhile, c.1008+1G>A has been identified as a hotspot variant among Chinese PDE patients (6). In this study, eight variants of the *ALDH7A1* gene were identified in six pediatric patients, including five missense variants and three splicing variants. Among these, three variants were reported for the first time, thereby expanding the variant spectrum of *ALDH7A1*. Two cases carried with the classic splicing variant: c.1008+1G>A. This variant is located at the boundary of exon 11 and intron, disrupting the 5′ splice donor GT signal, which leads to exon 12 skipping or abnormal splicing, ultimately resulting in protein inactivation. According to the ACMG guidelines, this variant is classified as pathogenic. Additionally, c.1061A>G and c.1547A>G were recurrently observed in this cohort and have been previously reported in Chinese PDE patients (17). Both variants are situated within the conserved functional domain of aldehyde dehydrogenase and are classified as likely pathogenic according to the ACMG guidelines. Based on the clinical presentation and inheritance pattern of the patient, we hypothesize that these two variants may be potential hotspot variants in Chinese PDE patients. However, children with the same variation exhibit significant variations in the age of onset, the type of seizures, the maintenance dose of pyridoxine, and developmental outcomes. This phenomenon might be associated with factors including allele combinations, modifier genes, and environmental influences (18). Future research should broaden the cohort and incorporate functional experiments alongside multi-omics analysis to clarify the underlying mechanisms.

Statistically, 64.53% of PDE cases begin within the first month of life (19). However, there have also been reports of cases with onset as late as after 1 year of age or even during adolescence (20). This indicates that the age of onset for this disease spans a wide range. A review of perinatal records revealed that PDE was associated with abnormal intrauterine fetal movements (33%), fetal distress (29%), respiratory distress (33%), and low Apgar scores (15%) (21). This led to some infants being diagnosed with hypoxic–ischemic encephalopathy at an early stage. Among the six cases in this group, three also exhibited fetal distress or respiratory distress. Therefore, for infants who develop refractory seizures shortly after birth and exhibit signs of perinatal hypoxia, it is important not to attribute the condition solely to hypoxic–ischemic encephalopathy and to remain highly vigilant for PDE. PDE presents with diverse seizure types, which may present as cluster seizures or have heat-sensitive characteristics. Some patients are prone to progressing to status epilepticus. Case 2 in this group initially presented during a febrile episode, manifesting as focal seizures. Subsequently, multiple febrile seizures and status epilepticus occurred, with normal or borderline VEEG backgrounds. Due to the high similarity of these clinical features to Dravet syndrome, vitamin B6 treatment was delayed. The above experience suggests that clinicians should include PDE in the differential diagnosis of heat-sensitive epilepsy and perform diagnostic pyridoxine testing as early as possible to reduce misdiagnosis and delay.

The EEG characteristics of PDE are highly heterogeneous. Early VEEG may present with hypsarrhythmia, focal or multifocal discharges, or even appear completely normal. As the condition progresses, some children who previously had no epileptiform discharges may also develop focal or multifocal discharges. In this group, all children experienced normalization of VEEG within days to months after regular and adequate treatment with pyridoxine. This indicates that vitamin B6 not only effectively controls seizures but also significantly reduces epileptiform discharges. Arntsen et al. (22) reported that outbreak suppression patterns are also common in PDE, but lack specificity. Although intravenous pyridoxine testing was once considered the gold standard for diagnosis, all four patients in this group experienced only temporary relief after administration. However, subsequent studies have shown that the neonatal response in cases of multisystem involvement may be delayed or less pronounced (23). Furthermore, VEEG improvement may also occur in non-PDE patients (24). Elevated levels of α-ALAS, P6C, and piperidic acid (PA) have been identified as potential newborn screening markers (10, 11), but they are not yet routinely used. Therefore, genetic testing remains the primary method for confirming PDE. It is recommended that whole-exome sequencing be performed as early as possible in infants who present with refractory seizures shortly after birth and have atypical VEEG findings.

Since Hunt et al. (1) first reported PDE in 1954, pyridoxine has become the first-line treatment for controlling its seizures (25, 26). All children in this group initially received 1 to 4 conventional antiseizure medication at the onset of the disease, but the efficacy was limited. Four cases received intravenous pyridoxine therapy during hospitalization. However, since the doses and types of conventional antiseizure medication were still being adjusted at the time, the efficacy could not be attributed solely to pyridoxine. After discharge, two patients did not continue oral pyridoxine therapy, leading to seizure recurrence. The other two patients achieved temporary efficacy with conventional antiseizure medication and did not promptly add pyridoxine. Considering the rarity of this condition and its atypical clinical presentation, we recommend the immediate initiation of intravenous pyridoxine therapy for unexplained neonatal or infantile early-onset epilepsy. Should this prove effective, oral maintenance therapy should be commenced post-discharge. In cases where conventional antiseizure medication yield only partial efficacy, PDE should not be dismissed. The 2021 International PDE Alliance guidelines recommend daily supplementation of 100 mg of pyridoxine for newborns, and a treatment dose of 15–30 mg/(kg·d) for infants (27). However, the guidelines also caution that long-term or high-dose (>500 mg) supplementation with vitamin B6 may result in reversible peripheral neuropathy. Considering age and side effects, the initial dose for this group was set at 7.7–12.5 mg/(kg·d). Except for one case that occasionally occurred during fever, all others achieved complete control. As age increased, the maintenance dose decreased to 3.3–7.8 mg/(kg·d). This suggests that some PDE patients only require low doses of pyridoxine to control seizures, and clinical adjustments should be individualized. The requirement for pyridoxine increases during infections, and seizures may worsen (28). It is recommended to temporarily increase the dose during acute fever. Regarding triple therapy, which involves supplementing with vitamin B6, arginine, and restricting lysine intake to improve neurodevelopmental outcomes, existing Level IV evidence does not show significant advantages (29, 30). The efficacy of triple therapy still requires further validation through high-quality studies.

Although pyridoxine is generally effective in controlling seizures in children with PDE, approximately 75% of patients still exhibit varying degrees of intellectual disability and developmental delay (31). Within this group, two patients who developed the disease in the neonatal period received regular vitamin B6 treatment within 3 months of age and were followed up until childhood, showing normal development. This suggests that early intervention may serve as a protective factor for long-term neurological development. Late-onset PDE is generally considered to have a better prognosis, potentially because of higher residual enzyme activity (32). However, the sole late-onset case in this group experienced moderate global developmental delay due to delayed diagnosis, frequent seizures, and delayed treatment. This underscores the necessity of assessing prognosis by considering both the age of onset and the timing of treatment. We discovered that among three cases of PDE with onset during the neonatal period and developmental delay, two families experienced recurrent miscarriages or had siblings who died prematurely due to seizures. This indicates that highly pathogenic *ALDH7A1* mutations may trigger fatal metabolic crises as early as the intrauterine period. The cranial MRI scans of the three aforementioned patients all indicated structural abnormalities, including subarachnoid enlargement, enlarged lateral ventricles and large occipital pools. Additionally, two of the three patients had a history of perinatal hypoxia. Large-scale studies have confirmed (33) that neonatal respiratory distress syndrome and abnormal brain development are both risk factors for motor development delay. However, early administration of vitamin B6 is a protective factor for language development. These findings confirm that the long-term developmental outcomes of PDE are influenced by a multifactorial interaction involving gene variant sites, perinatal hypoxia, age at onset, timing of treatment initiation, and brain structural abnormalities. Therefore, we recommend establishing a comprehensive management strategy encompassing prenatal diagnosis, neonatal screening, and early supplementation with pyridoxine to maximize the reduction of the risk of neurological sequelae.

## 5 Conclusion

This study identified eight *ALDH7A1* gene variants in six pediatric PDE patients. Three were reported internationally for the first time, expanding the known variant spectrum. The c.1061A>G and c.1547A>G variants were repeatedly seen, suggesting they may be hotspot variants in the Chinese population. Although early pyridoxine treatment can control seizures and reverse EEG abnormalities, patients’ clinical manifestations lack specificity, and there are significant differences in condition severity and neurodevelopmental outcomes. We recommend establishing a comprehensive management system including prenatal diagnosis, newborn screening, early pyridoxine intervention, and individualized maintenance therapy to improve prognosis. This study provides new evidence for PDE diagnosis and treatment. Future studies should expand the cohort to validate genotype – phenotype association.

## Data Availability

The original contributions presented in the study are publicly available. This data can be found here: https://data.mendeley.com/datasets/r8xpwr58vj/1. doi: 10.17632/r8xpwr58vj.1.
