# Supplementary figures and images for: Analysis of clinical features and genetic variants in Chinese children with pyridoxine-dependent epilepsy: a case series study
Source: Front Neurol. 2025 Sep 4;16:1609600. doi: 10.3389/fneur.2025.1609600 (PMC12443544; doi:10.3389/fneur.2025.1609600)

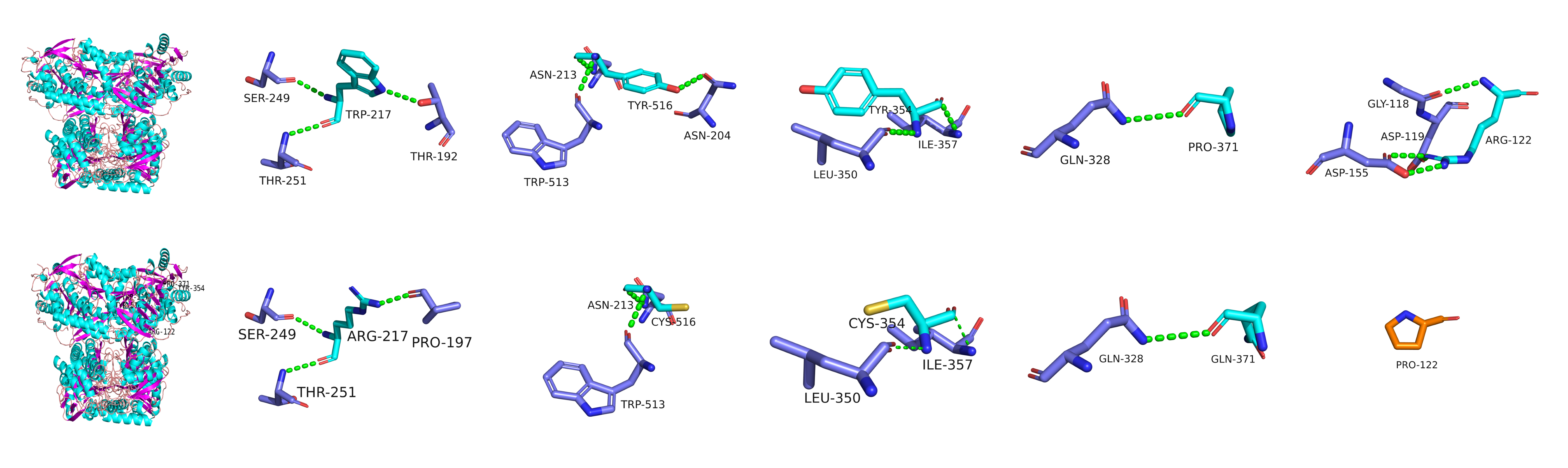

Supplement: Supplementary file 1 [file Image_1.png]

## Slide 1
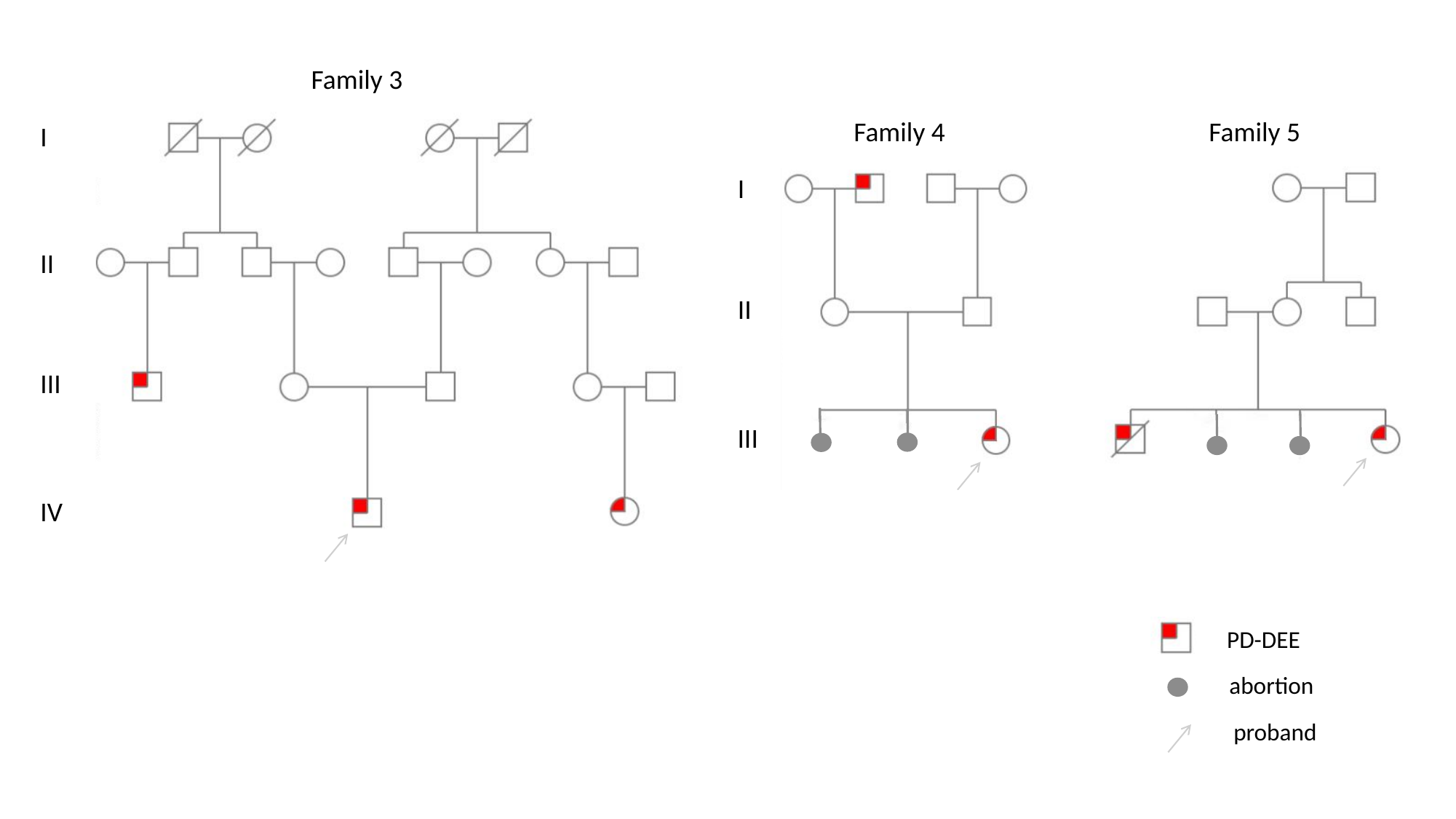

Family 3
I
II
III
IV
Family 4
I
II
III
Family 5
PD-DEE
abortion
proband

Supplement: Supplementary file 3 [file Presentation_2.pptx]
