# Supplementary material for: Analysis of clinical features and genetic variants in Chinese children with pyridoxine-dependent epilepsy: a case series study
Source: Front Neurol. 2025 Sep 4;16:1609600. doi: 10.3389/fneur.2025.1609600 (PMC12443544; doi:10.3389/fneur.2025.1609600)
Supplement: Supplementary file 2 [file Presentation_1.pptx]

## Slide 1
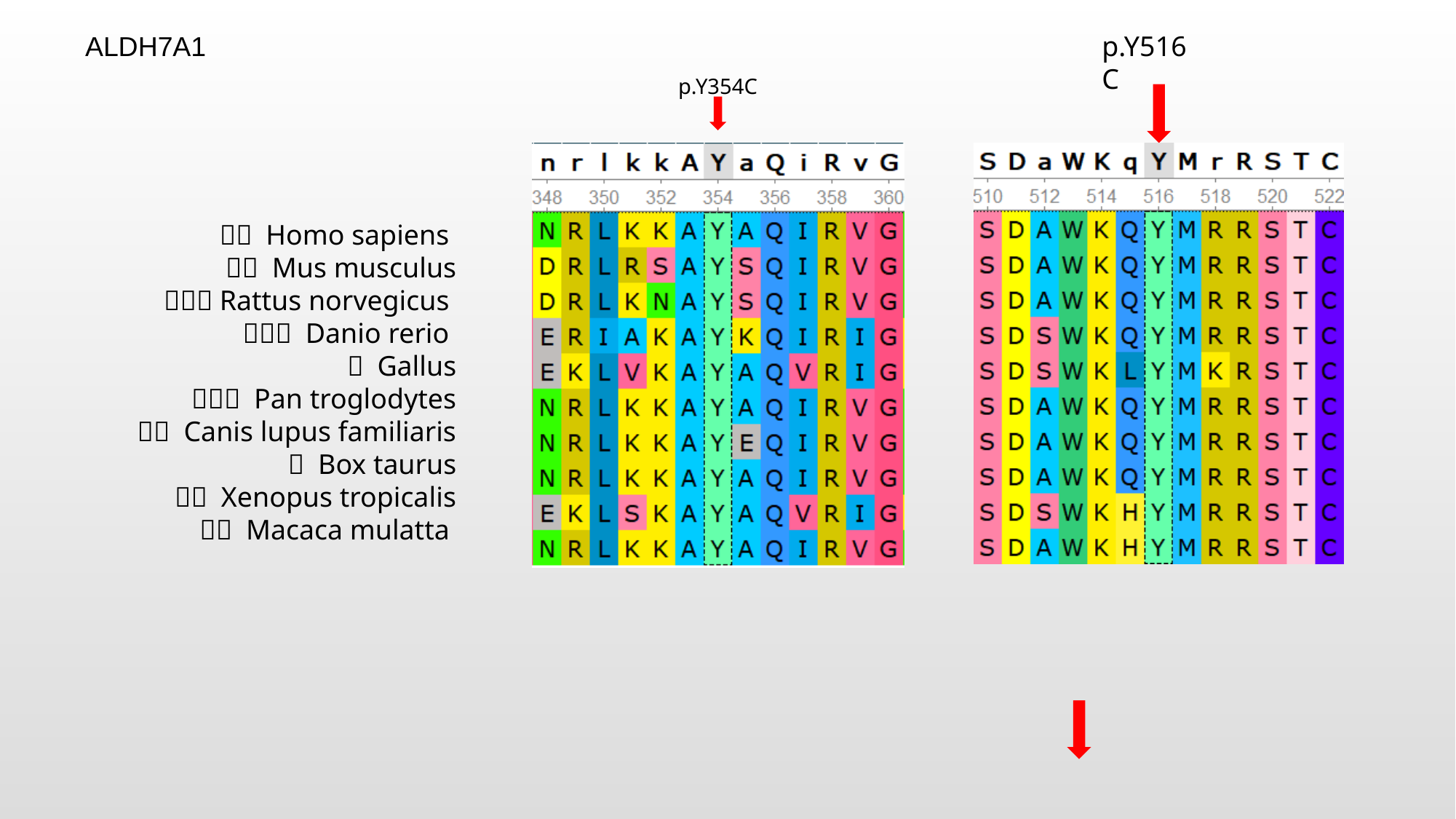

ALDH7A1
p.Y516C
p.Y354C
人类 Homo sapiens
家鼠 Mus musculus
褐家鼠Rattus norvegicus
斑马鱼 Danio rerio
鸡 Gallus
黑猩猩 Pan troglodytes
家犬 Canis lupus familiaris
牛 Box taurus
蟾蜍 Xenopus tropicalis
猕猴 Macaca mulatta

## Slide 2
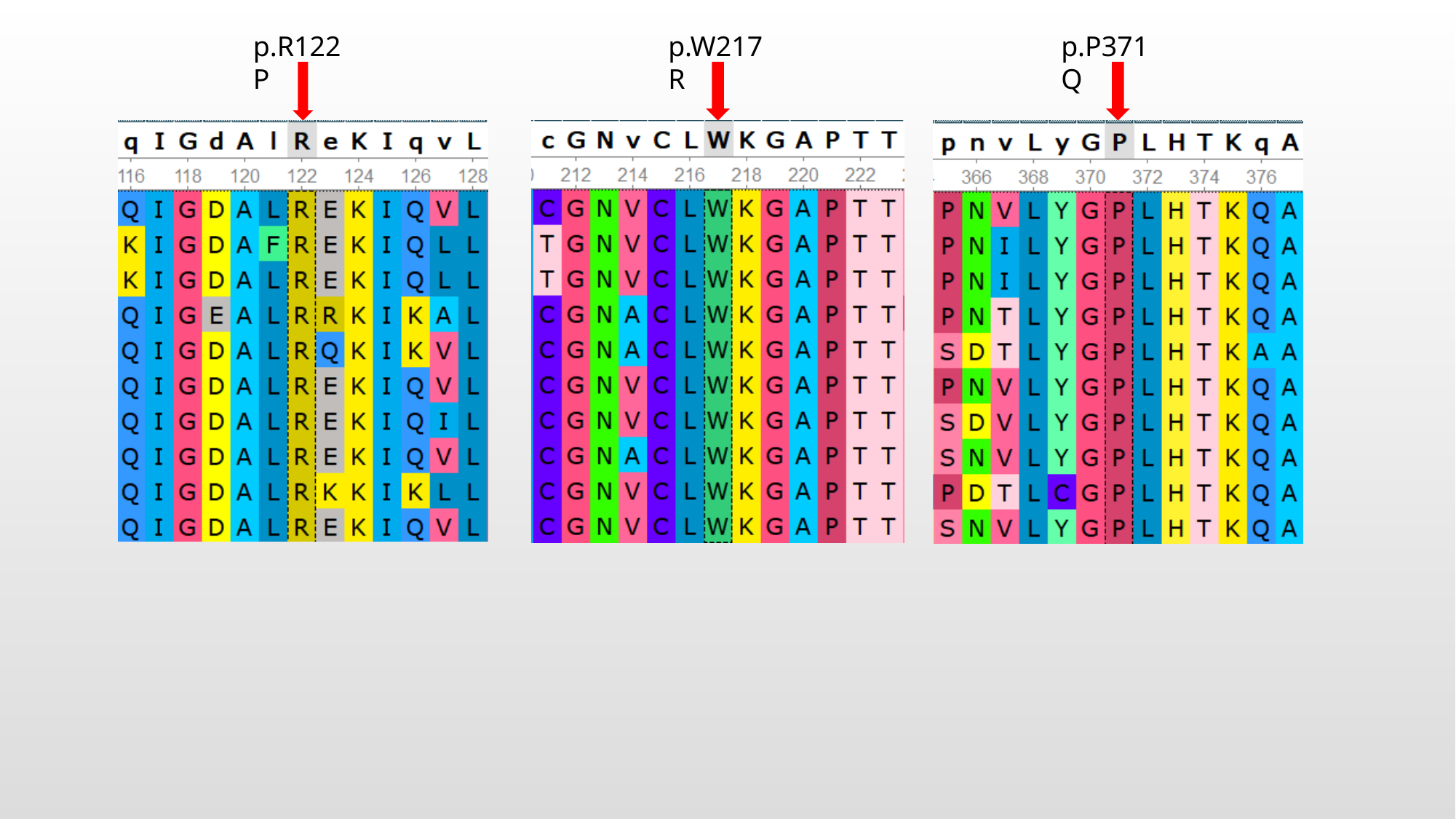

p.R122P
p.W217R
p.P371Q

## Slide 3
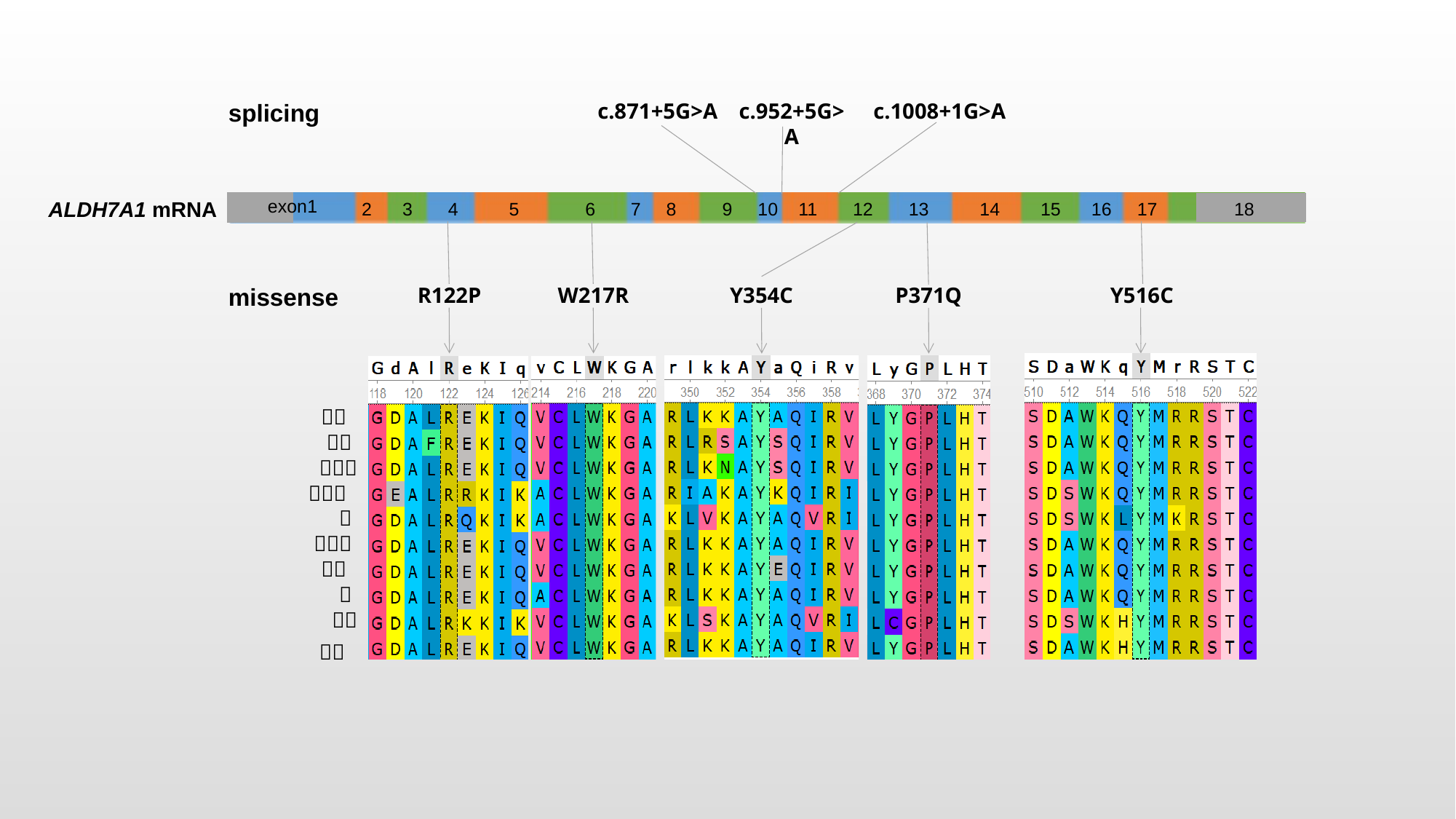

splicing
c.871+5G>A
c.952+5G>A
c.1008+1G>A
 ex 2 3 4 5 6 7 8 9 10 11 12 13 14 15 16 17 18
 exon1
 18
ALDH7A1 mRNA
missense
R122P
W217R
Y354C
P371Q
Y516C
人类
家鼠
褐家鼠
斑马鱼
鸡
黑猩猩
家犬
牛
 蟾蜍
猕猴

## Slide 4
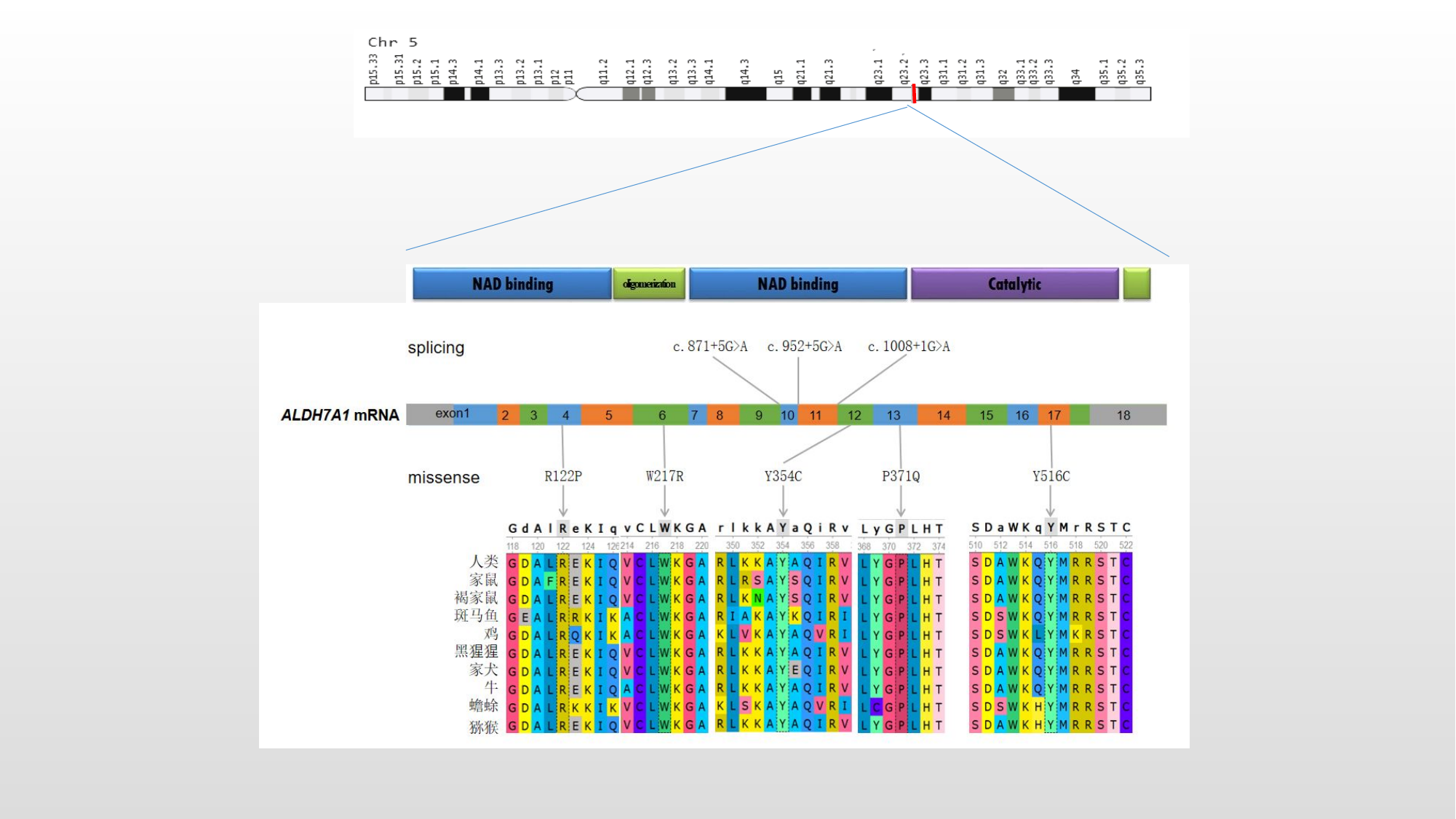

## Slide 5
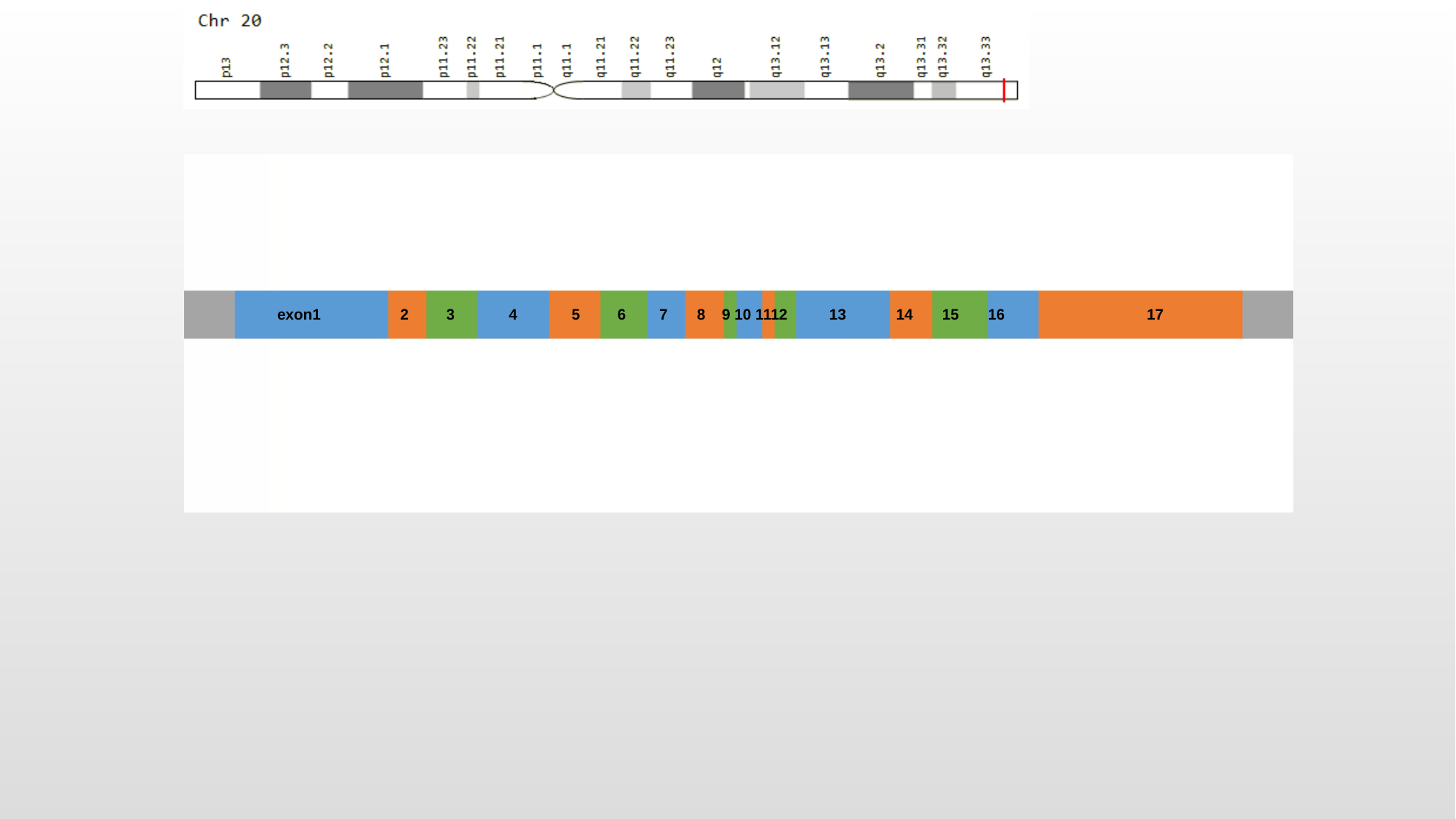

exon1 2 3 4 5 6 7 8 9 10 1112 13 14 15 16 17

## Slide 6
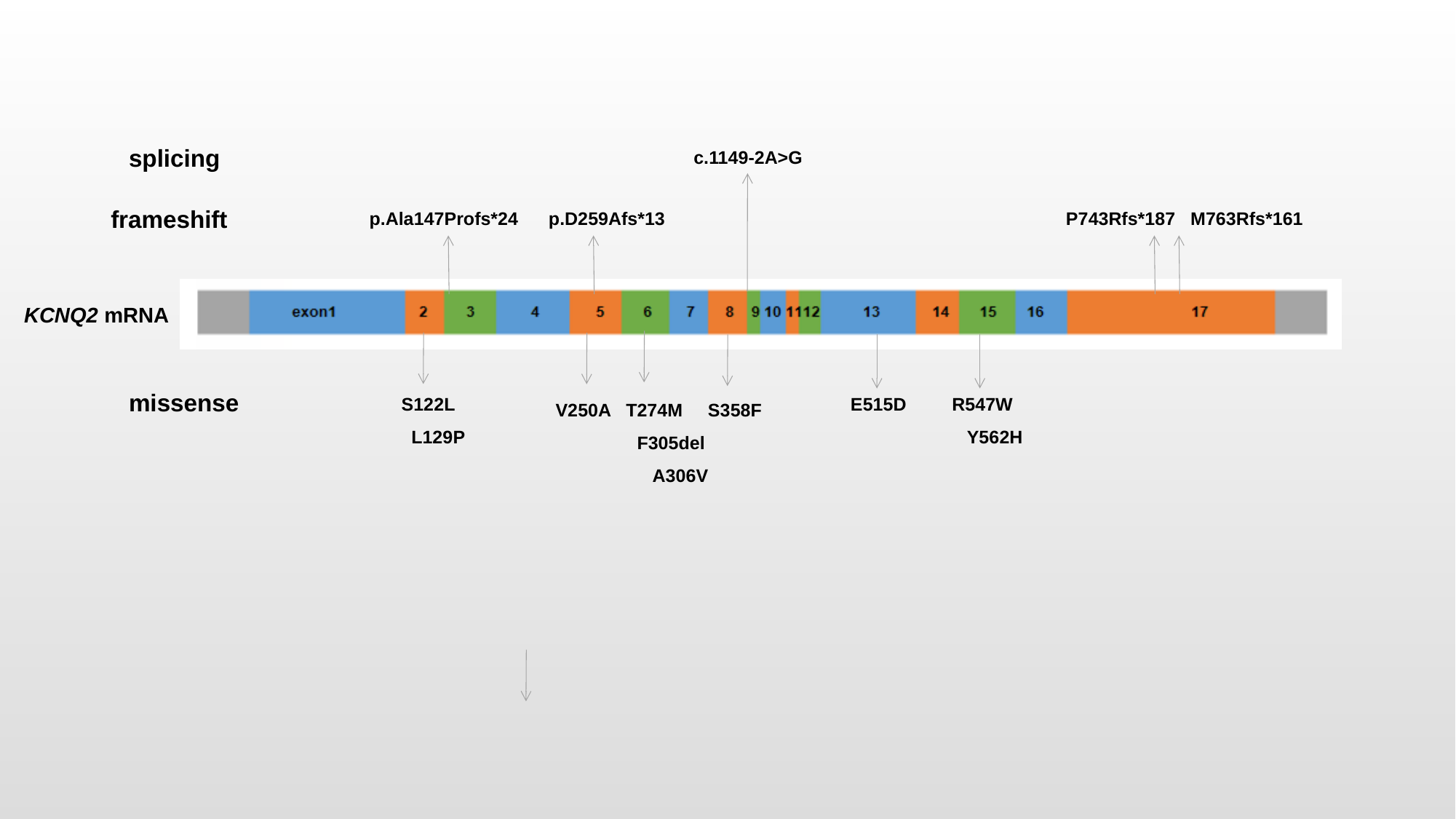

splicing
c.1149-2A>G
frameshift
 p.Ala147Profs*24 p.D259Afs*13 P743Rfs*187 M763Rfs*161
KCNQ2 mRNA
missense
V250A T274M S358F
 F305del
 A306V
 S122L E515D R547W
 L129P Y562H

## Slide 7
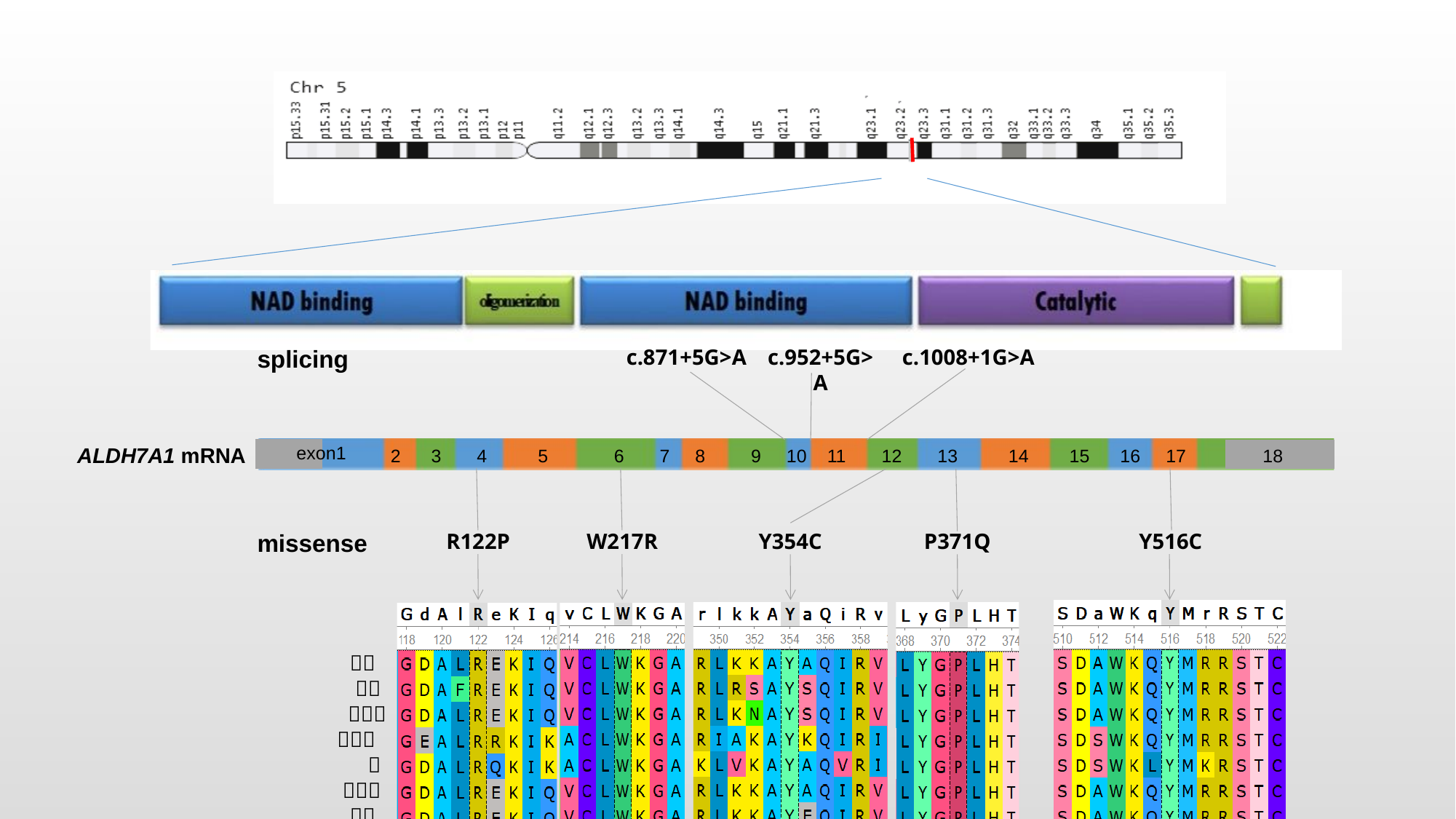

splicing
c.871+5G>A
c.952+5G>A
c.1008+1G>A
 ex 2 3 4 5 6 7 8 9 10 11 12 13 14 15 16 17 18
 exon1
 18
ALDH7A1 mRNA
missense
R122P
W217R
Y354C
P371Q
Y516C
人类
家鼠
褐家鼠
斑马鱼
鸡
黑猩猩
家犬
牛
 蟾蜍
猕猴
